# Sexting, Web-Based Risks, and Safety in Two Representative National Samples of Young Australians: Prevalence, Perspectives, and Predictors

**DOI:** 10.2196/13338

**Published:** 2019-06-17

**Authors:** Alyssa C Milton, Benjamin A Gill, Tracey A Davenport, Mitchell Dowling, Jane M Burns, Ian B Hickie

**Affiliations:** 1 Brain and Mind Centre University of Sydney Camperdown Australia

**Keywords:** youth, sexual behavior, cyberbullying, mental health, internet, safety, risk

## Abstract

**Background:**

The rapid uptake of information and communication technology (ICT) over the past decade—particularly the smartphone—has coincided with large increases in sexting. All previous Australian studies examining the prevalence of sexting activities in young people have relied on convenience or self-selected samples. Concurrently, there have been recent calls to undertake more in-depth research on the relationship between mental health problems, suicidal thoughts and behaviors, and sexting. How sexters (including those who receive, send, and two-way sext) and nonsexters apply ICT safety skills warrants further research.

**Objective:**

This study aimed to extend the Australian sexting literature by measuring (1) changes in the frequency of young people’s sexting activities from 2012 to 2014; (2) young people’s beliefs about sexting; (3) association of demographics, mental health and well-being items, and internet use with sexting; and (4) the relationship between sexting and ICT safety skills.

**Methods:**

Computer-assisted telephone interviewing using random digit dialing was used in two Young and Well National Surveys conducted in 2012 and 2014. The participants included representative and random samples of 1400 young people aged 16 to 25 years.

**Results:**

From 2012 to 2014, two-way sexting (2012: 521/1369, 38.06%; 2014: 591/1400, 42.21%; *P*=.03) and receiving sexts (2012: 375/1369, 27.39%; 2014: 433/1400, 30.93%; *P*<.001) increased significantly, not sexting (2012: 438/1369, 31.99%; 2014: 356/1400, 25.43%; *P*<.001) reduced significantly, whereas sending sexts (2012: n=35/1369, 2.56%; 2014: n=20/1400, 1.43%; *P*>.05) did not significantly change. In addition, two-way sexting and sending sexts were found to be associated with demographics (male, second language, and being in a relationship), mental health and well-being items (suicidal thoughts and behaviors and body image concerns), and ICT risks (cyberbullying others and late-night internet use). Receiving sexts was significantly associated with demographics (being male and not living with parents or guardians) and ICT risks (being cyberbullied and late-night internet use). Contrary to nonsexters, Pearson correlations demonstrated that all sexting groups (two-way, sending, and receiving) had a negative relationship with endorsing the ICT safety items relating to being careful when using the Web and not giving out personal details.

**Conclusions:**

Our research demonstrates that most young Australians are sexting or exposed to sexting in some capacity. Sexting is associated with some negative health and well-being outcomes—specifically, sending sexts is linked to suicidal thoughts and behaviors, body image issues, and ICT safety risks, including cyberbullying and late-night internet use. Those who do sext are less likely to engage in many preventative ICT safety behaviors. How the community works in partnership with young people to address this needs to be a multifaceted approach, where sexting is positioned within a wider proactive conversation about gender, culture, psychosocial health, and respecting and caring for each other when on the Web.

## Introduction

### Background

In recent decades, the increasing number of young people engaging in sexting has become a highly publicized and controversial part of the information and communication technology (ICT) transformation [1]. Although the definition of sexting varies, it refers to the sending, receiving, or forwarding of sexually explicit images, videos, or messages [2]. Sexting may represent a normal expression of sexuality among young people [3], with some researchers highlighting that sexting may be “the new first base” [4]. Despite this, it attracts concern from parents, teachers, policy makers, and organizations working with young people [5]. This concern often stems from sexting being linked to legal consequences [6,7] when legal sexting provisions for minors do not apply [8]—as well as negative social, emotional, and mental health effects [1]. Given the likely relationship between sexting and mental health problems, comprehensive research using random and representative national samples is needed to improve our understanding of the prevalence, beliefs, and associations of sexting to inform support practices and educational efforts targeting young people. In addition, outcomes of such research may help to inform mental health prevention and early intervention efforts targeted at young people, which are key priorities for the Australian government [9] and internationally [10].

### Sexting Prevalence

International research has primarily focused on sexting prevalence among adolescents and young adults; however, this has yielded broad variability in results. Recent systematic review evidence suggests that internationally, only a minority of young people engage in sexting—with an average of 1 in 7 sending sexts and 1 in 4 receiving sexts (which varies by age, reporting year, and method of sexting) [2]. Within the Australian context, the prevalence estimates are higher and remain variable, particularly for those who have received sexts. Specifically, 43% to 49% of young people report sending sexts, 42% to 67% report that they have received sexts, and 40% to 46% report having sent or received sexts [6,7,11,12]. Variability in results is at least partially attributed to inconsistencies in sampling techniques [1,13]. For example, all previous Australian studies reporting prevalence have employed convenience or self-selected sampling techniques. Research that applies nationally representative and random sampling survey techniques is clearly needed.

Variability in prevalence rates has been partially explained by inconsistencies in definitions and measurement of sexting behaviors [1,13,14]. Previous Australian research has measured the lifetime experience of sexting, as opposed to sexting frequency over a specific period. This measurement approach poses challenges when comparing adolescent with young adult sexting prevalence, as young adults will have had more opportunities to engage in sexting. Another common issue when considering sexting prevalence in Australia is that measures of sending and receiving sexts have frequently been presented as 1 sexting variable. This creates challenges in comparing sexting prevalence and correlates between studies, either nationally or internationally. Addressing these issues relating to the operationalization and measurement of sexting warrants focus.

### Sexting Predictors and Correlates

Multiple sociodemographic, mental health, and well-being variables have been implicated in the sexting literature [1]. Systematic review evidence suggests that compared with children and adolescents, young adults have higher prevalence rates for sending and receiving sexts [1]. However, most findings relating to sociodemographics are inconsistent, with ethnicity, gender, sexual orientation, education level, and employment status all yielding mixed findings.

Mental health and well-being variables and their association with sexting produce similar mixed findings. Sexting has been found to be associated with substance misuse [15], mental health problems [6], and suicidal thoughts and behaviors [16]. However, other studies report no associations between sexting and depression, anxiety, self-esteem [17], or mental health problems [18,19]. Poorer biopsychosocial well-being in young people results from sexting in combination with cyberbullying [20]. Moreover, young people who engage in sexting are more likely to not only experience cyber victimization but also to be victimized by different types of cyber victimization [21]. Despite the number of individual studies looking at sexting and its correlates, in the Australian context, there is a distinct need for a comprehensive examination of the associations between different types of sexting—such as sending, receiving, two-way, and not sexting—and other factors including a young person’s sociodemographics, mental health and well-being, and other ICT risk behaviors such as cyberbullying.

### Sexting and Information and Communication Technology Use

Research examining technology use and sexting has reported associations between sexting and time spent texting [16], problematic smartphone use [22], having a Facebook account, and Web-based video chatting with strangers [23]—but not hours spent on the internet daily [17]. Sexting predominately occurs through smartphone apps, such as Snap Inc.‘s Snapchat—with these apps being perceived by participants as a more “...convenient, safe, and informal means of sexting communication than other mediums, such as e-mail or Facebook, regardless of the actual risk of unauthorized distribution” [24]. How young Australians practice ICT safety when using such technologies and forming an understanding of how ICT safety practices relate to different types of sexting behaviors (ie, sending, receiving, two-way, and not sexting) has not, to our knowledge, been researched.

### This Research

This study extends the Australian literature to provide sexting prevalence and correlates among young Australians using the results from the 2012 and 2014 Young and Well National Surveys, which include representative and random samples of 1400 young people aged 16 to 25 years. This research directly addressed the research gaps within the Australian context, including the reliance on convenience or self-selected samples, and the lack of comprehensive reporting on the relationship between sexting and other factors such as sociodemographics, mental health and well-being items, and ICT risks and ICT safety practices.

This study has 4 main aims, including the assessment of (1) the changes in the frequency of young people’s sexting activities from 2012 to 2014; (2) young people’s beliefs about sexting; (3) the association of demographics, health and well-being items, and internet use with sexting activity; and (4) the relationship between sexting and ICT safety skills.

## Methods

### Participants and Recruitment

This study received ethics approval from The University of Sydney human research ethics committee (2012 Protocol No. 2012/1640; 2014 Protocol No. 2014/741) and was a partnership between the authors who were associated with the Young and Well Cooperative Research Centre (CRC; 2011-16) and The University of Sydney’s Brain and Mind Centre (BMC). The survey was run using computer-assisted telephone interviewing (CATI). These telephone interviews were conducted by an independently contracted company—the Social Research Centre (Melbourne, Victoria)—that was commissioned by the authors to run the CATI. Respondents were randomly selected using random digit dialing (RDD) and included 700 young men and 700 young women aged 16 to 25 years. RDD has been cited as the historical gold standard for population-based control recruitment when conducting epidemiologic research [25]. Stratification was used to ensure that the samples were representative of the general population in terms of age, gender, and geographic location across all Australian states and territories. Participation was voluntary, and verbal consent was obtained at the start of the telephone interview. The respondents were excluded if they had English language difficulties or if they were uncomfortable with the interview being conducted in English. For all respondents aged 16 or 17 years, consent from a coresident parent or guardian was sought in addition to the young person’s consent before the commencement of the survey. Research was conducted in accordance with the Social Research Centre’s code of practice, and the survey took approximately 20 min to complete.

### Questionnaire

The first and second Young and Well National Surveys (2012 and 2014) included questions relating to demographics, mental health and well-being, health perceptions of Australian youth, use of the internet, Web-based and communication risks (digital abuse such as bullying and sexting), digital literacy, and ICT safety skills.

Mental health and well-being survey items included the following: (1) the Kessler Psychological Distress Scale [26] assessing psychological distress during the past month; (2) the Psychiatric Frequency Symptom Scale (suicidality subscale) [27] measuring suicidal thoughts and behaviors; (3) experience of a mental health diagnosis and an alcohol or other substance use problem were measured with 2 single items asking an individual’s desire to cut back and social/professional encouragement to stop; (4) issues of personal concern items included alcohol, body image, bullying or emotional abuse, coping with stress, depression, drugs, and self-harm; (5) resilience was measured by the Brief Resilience Coping Scale [28]; and (6) perceived social support and conflict in close relationships were measured in the 2014 survey only by the Social Support and Conflict Scale [29].

Internet use was based on survey items used in the *headspace* National Youth and Parent Community Survey [30]. Items of interest included (1) average time spent on the internet and (2) late-night internet use after 11 pm. Furthermore, respondents were asked about “Internet rules that some people follow” (2012 National Survey only) and personal experience of applying ICT safety (2014 National Survey only). Web-based and communication risks items were developed by the research group based on the reviews of national and international literature. To determine sexting beliefs and behaviors, respondents were asked (1) whether they considered sexting a serious problem for young people, (2) whether they had seen or received images or messages of a sexual nature in the previous 12 months, (3) whether they had sent messages or images of a sexual nature in the last 12 months, and (4) reasons for sexting and beliefs about sexting adapted from the study by Henderson [31] (2012 National Survey only). For the cyberbullying component, participants were asked (1) whether they considered sexting a serious problem for young people, (2) the frequency for which they had been cyberbullied in the previous 12 months, and (3) the frequency for which they had cyberbullied others in the previous 12 months.

### Analysis

Data were analyzed using SPSS 22.0 for Windows [32]. Univariate descriptive and frequency statistics were used to describe all demographic, clinical, and internet use items. Chi-square analysis assessed changes in sexting activity rates from 2012 to 2014. Phi was used to determine the effect size. Frequency statistics were used to describe young people’s beliefs about sexting. Binary logistic regression analyses were used to investigate possible predictors of sexting (2 way, sending, receiving, and no sexting), reported as adjusted odds ratios (AORs) at a 95% confidence interval (CI). Pearson bivariate correlations determined the relationship between sexting groups (2way, sending, receiving, and no sexting) and ICT safety items. No missing data were imputed for any analysis.

## Results

### Sample Characteristics

In both 2012 and 2014, half of the 1400 young people who participated were male (2012: 700/1400, 50.00%; 2014: 705/1400, 50.36%). Approximately one-third of the respondents were from each target age group: 16 to 18 years (2012: 484/1400, 34.57%; 2014: 499/1400, 35.64%), 19 to 21 years (2012: 466/1400, 33.28%; 2014: 464/1400, 33.14%), and 22 to 25 years (2012: 450/1400, 32.14%; 2014: 437/1400, 31.21%). In both surveys, the majority (2012: 1086/1400, 77.57%; 2014: 1107/1400, 79.07%) of the respondents did not speak any language other than English. Rates of identification with Aboriginal or Torres Strait Islander descent (2012: 30/1400, 2.14%; 2014: 48/1400, 3.43%) were reflective of national census rates [33]. The majority of young people lived in major cities (2012: 1047/1400, 74.79%; 2014: 1048/1400, 74.86%) and lived with at least one of their parents or guardians (2012: 1031/1400, 73.64%; 2014: 1057/1400, 75.50%). In addition, over half of them described education as their main current activity (2012: 869/1399, 58.18%; 2014: 814/1398, 57.79%) and one-third of respondents were employed in some capacity (2012: 493/1399, 35.24%; 2014: 474/1398, 33.86%). Changes in sociodemographics, mental health and well-being, and internet use between 2012 and 2014 are presented in Multimedia Appendix 1.

### Sexting Prevalence

Young people’s overall sexting activities from 2012 to 2014 changed significantly; a breakdown of these changes by type of sexting activity is presented in Table 1. From 2012 to 2014, young people most commonly endorsed being reciprocal two-way sexters (2012: 521/1369, 38.06%; 2014: 591/1400, 42.21%), and this increased significantly from 2012 to 2014 (*P*=.03). The number of young people reporting only receiving sexts increased significantly from 2012 to 2014 (2012: 375/1369, 27.39%; 2014: 433/1400, 30.93%; *P*<.001), whereas those only sending sexts did not change significantly over time and was the least endorsed sexting behavior (2012: 35/1369, 2.56%; 2014: 20/1400, 1.43%). The proportion of young people reporting that they were a nonsexter reduced significantly from one-third to a quarter of respondents between 2012 and 2014 (2012: 438/1369, 31.99%; 2014: 356/1400, 25.43%; *P*<.001).

A more detailed breakdown of the specific types of sexting activities that young people engaged in is presented in Table 2.

**Table 1:** Prevalence of sexting between 2012 and 2014.

Sexting category	Yes in 2012 (N=1369), n (%)	Yes in 2014 (N=1400), n (%)	Chi-square (*df*)	*P* value	Phi
Two-way sexting	521 (38.06)	591 (42.21)	5.0 (1)	.03	.04
Only sending sexts	35 (2.56)	20 (1.43)	2.6 (1)	.11	−.03
Only receiving sexts	375 (27.39)	433 (30.93)	19.3 (1)	<.001	.08
Nonsexter	438 (31.99)	356 (25.43)	14.6 (1)	<.001	-.07

**Table 2:** Frequency of young people’s sexting behaviors.

Sexting behavior		Yes in 2012 (N=1369), n (%)	Yes in 2014 (N=1400), n (%)	Chi-square (*df*)	*P* value	Phi
**Receiver^a^**						
	Been sent sexual message	552 (40.32)	644 (46.00)	9.1 (1)	.003	.057
	Seen a sexual message posted where others could see it	490 (35.79)	598 (42.71)	13.9 (1)	<.001	.104
	Seen other people perform acts of a sexual nature	361 (26.37)	540 (38.57)	47.0 (1)	<.001	.130
	Been asked to talk about acts of a sexual nature with someone	365 (26.66)	489 (34.93)	22.2 (1)	<.001	.089
	Been asked for a photo or video showing yourself nude or nearly nude	266 (19.43)	435 (31.07)	49.6 (1)	<.001	.134
	None	473 (34.55)	376 (26.86)	19.3 (1)	<.001	−.083
	Refused	1 (0.07)	4 (0.29)	1.7 (1)	.19	.025
**Sender^a^**						
	None	813 (59.39)	789 (56.36)	2.6 (1)	.11	-.107
	Talked about acts of a sexual nature with someone	465 (33.97)	513 (36.64)	2.2 (1)	.14	.028
	Sent someone a sexual message	398 (29.07)	449 (32.07)	2.9 (1)	.09	.033
	Sent someone a photo or video showing yourself nude or nearly nude	140 (10.23)	237 (16.93)	26.4 (1)	<.001	.098
	Asked someone for a photo or video showing themselves nude or nearly nude	122 (8.91)	170 (12.14)	7.7 (1)	.006	.053
	Posted a sexual message posted where others could see it	41 (2.99)	24 (1.71)	5.0 (1)	.03	−.042
	Refused	2 (0.15)	8 (0.57)	3.5 (1)	.06	.062

^a^Arranged from most to least commonly endorsed item by receiver and by sender from the 2014 data*.*

In both 2012 and 2014, when asked about the type of sexts being received, the most frequently reported item was being sent a sexual message (2012: 552/1369, 40.32%; 2014: 644/1400, 46.00%). Overall, the rates of endorsement of items relating to being a receiver of sexts increased significantly between 2012 and 2014 across all items, whereas endorsement of none fell significantly. Significant increases in endorsement rates between 2012 and 2014 for 4 items relating to the type of sexts being sent were found (sent someone a sexual message, sent someone a photo or video showing yourself nude or nearly nude, asked someone for a photo or video showing themselves nude or nearly nude, and posted a sexual message posted where others could see it). However, for all respondents, the most commonly endorsed item relating to sending sexts was none (2012: 813/1369, 59.39%; 2014: 789/1400, 56.36%).

### Beliefs About Sexting

Consistently in both 2012 and 2014, over half (2012: 691/1369, 50.47%; 2014: 707/1268, 50.50%) of the respondents thought sexting was a serious problem for young people (χ^2^=1.2; *P*=.27). The reasons for sexting and beliefs about sexting were explored in the 2012 survey and are presented in Table 3 and 4, respectively. The most commonly reported reason for sexting was “to get attention from a dating partner” (1217/1369, 88.90%). Sexting causing “serious negative consequences” was endorsed by nearly all respondents (1263/1369, 92.26%), and the vast majority believed that “messages usually end up being seen by more than just those to whom they were sent” (1157/1369, 84.51%).

### Sexting Predictors

The associations between sexting activity and demographic, health and well-being items, and internet use were examined. AORs for the 2014 data are presented in Table 5. Additional crude risk ratios showing the strength of the relationships between each variable and sexting behavior are presented in Multimedia Appendix 2.

**Table 3:** Young people’s reasons sexting in 2012 (N=1369).

Item		Yes, n (%)
**Reasons that young people send or post sexual material^a,b^**		
	To get attention from a dating partner	1217 (88.90)
	To be fun and flirtatious	1111 (81.15)
	To be sexy or initiate sexual activity	1093 (79.84)
	They feel pressured to by friends or a dating partner	959 (70.05)
	A form of self-expression	677 (49.45)
	Don’t know	22 (1.61)
	Other reason	9 (0.66)

^a^Items not asked in 2014.

^b^Arranged from most to least commonly endorsed item.

**Table 4:** Young people’s beliefs about sexting in 2012 (N=1369).

Item		Agree/strongly agree, n (%)
**Beliefs about sexting^a,b^**		
	It can cause serious negative consequences	1263 (92.26)
	Messages usually end up being seen by more than just those to whom they were sent	1157 (84.51)
	Females have to worry about messages being viewed by someone other than the person they had originally intended it for, more than males do	1067 (77.94)
	There is pressure among young people to sext	824 (60.19)
	Males have to worry about messages being viewed by someone other than the person they had originally intended it for, more than females do	413 (30.17)
	It’s no big deal	359 (26.22)

^a^Items not asked in 2014.

^b^Arranged from most to least commonly endorsed item.

**Table 5:** Adjusted odds ratios of the association of demographics, health and well-being items, and internet use with sexting activity (2014; N=597).

Variable			Sexting activity versus all others			
			Two-way	Sender^a^	Receiver^b^	No sexting
**Demographics**						
	**Gender (male vs female)**					
		AOR Exp (B)^c^	*0.63* ^d^	*0.58* ^d^	*0.59* ^d^	*1.73* ^d^
		95% CI^e^	*0.41-0.96*	*0.38-0.89*	*0.37-0.95*	*1.07-2.80*
	**Age (16-18 years vs 19-21 years)**					
		AOR Exp (B)	1.13	1.11	0.83	1.22
		95% CI	0.71-1.80	0.70-1.76	0.51-1.37	0.73-2.04
	**Age (16-18 years vs 22-25 years)**					
		AOR Exp (B)	1.34	1.23	0.72	1.56
		95% CI	0.78-2.30	0.72-2.10	0.41-1.27	0.87-2.79
	**English only language spoken (no vs yes)**					
		AOR Exp (B)	*1.66* ^d^	*1.71* ^d^	1.53	0.62
		95% CI	*1.01-2.73*	*1.04-2.80*	0.93-2.55	0.37-1.03
	**Indigenous (no vs yes)**					
		AOR Exp (B)	0.74	0.93	0.93	0.7
		95% CI	0.23-2.37	0.30-2.94	0.23-3.80	0.14-3.65
	**Location (major city vs regional, rural, or remote)**					
		AOR Exp (B)	1.01	1	0.97	1.05
		95% CI	0.66-1.56	0.65-1.54	0.59-1.59	0.63-1.74
	**Currently in education (no vs yes)**					
		AOR Exp (B)	0.72	0.8	0.66	1.32
		95% CI	0.36-1.42	0.41-1.59	0.29-1.50	0.58-3.02
	**Currently in employment (no vs yes)**					
		AOR Exp (B)	1.36	1.57	1.05	0.76
		95% CI	0.68-2.74	0.78-3.16	0.45-2.45	0.32-1.81
	**Live with at least one parent or guardian (no vs yes)**					
		AOR Exp (B)	0.88	0.79	*0.53* ^d^	*2.36* ^f^
		95% CI	0.54-1.44	0.48-1.28	*0.29-0.93*	*1.28-4.36*
	**Currently in a relationship (no vs yes)**					
		AOR Exp (B)	*2.16* ^g^	*2.11* ^g^	1.14	0.9
		95% CI	*1.47-3.19*	*1.43-3.09*	0.73-1.76	0.57-1.41
**Health and well-being**						
	**Psychological distress (K10^h^: low/moderate vs high/very high)**					
		AOR Exp (B)	0.68	0.67	1.36	0.72
		95% CI	0.39-1.17	0.39-1.15	0.74-2.49	0.39-1.35
	**Suicidal ideation and or acts (PSFS^i^)**					
		AOR Exp (B)	*1.86* ^d^	*2.21* ^d^	1.1	0.63
		95% CI	*1.00-3.46*	*1.19-4.10*	0.53-2.33	0.28-1.41
	**Mental health diagnosis (no vs yes)**					
		AOR Exp (B)	0.92	0.93	1.68	0.57
		95% CI	0.57-1.51	0.57-1.52	0.96-2.96	0.32-1.02
	**Alcohol or other substance misuse diagnosis (no vs yes)**					
		AOR Exp (B)	3.74	3.34	0.72	1.96
		95% CI	0.38-36.96	0.33-33.52	0.07-7.76	0.17-21.99
	**Personal concern: Alcohol (no vs yes)**					
		AOR Exp (B)	1.04	1	1.04	1.02
		95% CI	0.58-1.87	0.56-1.78	0.51-2.11	0.49-2.09
	**Personal concern: Body Image (no vs yes)**					
		AOR Exp (B)	*2.06* ^f^	*2.00* ^f^	1.44	0.7
		95% CI	*1.33-3.19*	*1.30-3.08*	0.90-2.32	0.43-1.14
	**Personal concern: Bullying (no vs yes)**					
		AOR Exp (B)	1.37	1.16	0.85	1.53
		95% CI	0.80-2.34	0.68-1.97	0.46-1.58	0.81-2.89
	**Personal concern: Stress (no vs yes)**					
		AOR Exp (B)	1.11	1.16	1.26	0.75
		95% CI	0.71-1.73	0.74-1.81	0.78-2.04	0.46-1.22
	**Personal concern: Depression (no vs yes)**					
		AOR Exp (B)	0.63	0.68	0.62	1.56
		95% CI	0.35-1.12	0.38-1.20	0.33-1.16	0.81-2.99
	**Personal concern: Drugs (no vs yes)**					
		AOR Exp (B)	1.85	2	2.39	*0.35* ^d^
		95% CI	0.91-3.75	0.99-4.05	0.98-5.83	*0.14-0.90*
	**Personal concern: Self-harm (no vs yes)**					
		AOR Exp (B)	0.63	0.62	0.72	1.45
		95% CI	0.31-1.25	0.31-1.24	0.32-1.64	0.61-3.43
	**Resilience (BRCS^j^)**					
		AOR Exp (B)	1.03	1.03	1.02	0.99
		95% CI	0.96-1.12	0.95-1.11	0.94-1.11	0.90-1.08
	**Social support (SSCS^k^)**					
		AOR Exp (B)	0.96	0.96	1.03	0.97
		95% CI	0.88-1.04	0.88-1.04	0.93-1.13	0.88-1.07
**Internet use and Web-based communication risks**						
	**Has been cyberbullied in the past 12 months (no vs yes)**					
		AOR Exp (B)	1.11	1.07	*4.61* ^f^	*0.22* ^f^
		95% CI	0.61-2.02	0.59-2.94	*1.85-11.50*	*0.09-0.56*
	**Cyberbullied others in the past 12 months (no vs yes)**					
		AOR Exp (B)	*5.28* ^g^	*4.79* ^g^	1.4	0.87
		95% CI	*2.20-12.65*	*2.00-11.44*	0.41-4.83	0.25-3.02
	**Average time spent on internet**					
		AOR Exp (B)	1.02	1.02	0.98	1.02
		95% CI	0.96-1.09	0.96-1.09	0.92-1.05	0.95-1.09
	**Late-night internet use (no vs yes)**					
		AOR Exp (B)	*1.92* ^f^	*1.88* ^f^	*2.58* ^g^	*0.38* ^g^
		95% CI	*1.24-2.95*	*1.23-2.88*	*1.67-3.98*	*0.24-0.59*

^a^Includes all respondents who reported sending sexts in any form.

^b^Includes all respondents who reported receiving sexts in any form.

^c^AOR Exp (B): adjusted odds ratio exponentiation of the B coefficient.

^d^Correlation is significant at the .05 level (2-tailed). Significant items are italicized in the table.

^e^95% Confidence Interval.

^f^Correlation is significant at the .01 level (2-tailed). Significant items are italicized in the table.

^g^Correlation is significant at <.001 (2-tailed). Significant items are italicized in the table.

^h^Kessler psychological distress scale.

^i^Psychiatric Frequency Symptom Scale (suicidality subscale).

^j^Brief Resilience Coping Scale.

^k^Schuster’s Social Support and Conflict Scale.

Two-way sexting and sending sexts yielded similar results. Specifically, being male (two-way: *P=*.01; sender: *P=*.01), speaking any language other than English (two-way: *P=*.046; sender: *P=*.03), being in a relationship (two-way: *P*<.001; sender: *P*<.001), experiencing suicidal thoughts and behaviors (two-way: *P=*.048; sender: *P=*.012), reporting body image concerns (two-way: *P*=.001; sender: *P=*.002), cyberbullying others (two-way: *P*<.001; sender: *P*<.001), and late-night internet use (two-way: *P*=.003; sender: *P=*.004) were associated with significantly greater AORs of both two-way sexting and sending sexts. Receiving sexts was significantly associated with being male (*P=*.03), being cyberbullied (*P=*.001), late-night internet use (*P*<.001), and lower rates of living with parents or guardians (*P=*.03).

Not sexting was significantly associated with being female (*P=*.03) and living with parents or guardians (*P=*.006) and lower rates of drugs being a personal concern (*P=*.03), being cyberbullied (*P=*.001), and late-night internet use (*P<*.001).

### Sexting and Information and Communication Technology Safety Skills

Sexting and its relationship with ICT safety items were measured with bivariate correlations. As presented in Table 6, all sexting activities (two-way, sending and receiving) had a significantly positive relationship with respondents “removing content they had posted online” (*P*<.001) and “sharing particular kinds of information about themselves so that people won’t ask them about their real feelings or desires” (*P*<.001). All sexting activities (two-way *P*<.001, sending *P*<.001, and receiving *P*=.002) had a negative relationship with endorsing that people should not give out a personal address or phone number. Sending sexts (*P*=.002) and two-way sexting (*P*=.002) were negatively correlated with endorsing that people should keep their computer in a public room and being careful with what one posts online. Being a receiver of sexts demonstrated a strong positive correlation with reporting a person or incident to a site master (*P*<.001) and ignoring threatening or offensive behavior toward both themselves (*P*<.001) and others (*P*=.003). Being a nonsexter was significantly negatively correlated with not posting because of future concerns (*P*=.02), removing content (*P*<.001), limiting information shared (*P*=.009), reporting others (*P*<.001), and ignoring threatening or offensive behavior toward themselves (*P*<.001) or others (*P*=.007). However, nonsexters were more likely to endorse not using a real name (*P*=.04), not giving out an address or phone number (*P*<.001), and being careful with what one should post online (*P*=.009).

**Table 6:** Bivariate correlations between sexting and information and communication technology safety items.

Information and communication technology safety items		Sexting activities, Pearson *r*			
		Two-way	Sending^a^	Receiving^b^	Nonsexter
**Have you ever done any of the following^c^**					
	Used the profile settings on your online profiles to protect your privacy and security (N=670)	−.001	−.015	.024	−.012
	Limited what certain friends or community members can or cannot see (N=671)	.046	.042	.041	−.037
	Decided not to post something online because you were concerned it might reflect badly on you in the future (N=669)	.066	.063	.099^d^	−.087^d^
	Tried to remove content you posted online (N=668)	.150^e^	.143^e^	.173^e^	−.168^e^
	Taken steps to try to limit the amount of information available about you on the Internet (N=673)	.003	−.001	.104^f^	−.101^f^
	If you’ve seen someone being cruel or mean online, looked for or asked someone for advice about what to do (N=667)	.025	.018	.053	−.046
	Reported a person or incident to a site master (N=672)	.064	.059	.219^e^	−.217^e^
	Reported a person or incident to an authority, eg, a teacher, police (N=674)	−.021	−.022	.090^d^	−.090^d^
	Ignored threatening or offensive behavior toward you (N=668)	.083^d^	.069	.167^e^	−.155^e^
	Ignored threatening or offensive behavior toward someone else (N=663)	.091^d^	.080^d^	.114^f^	−.104^f^
	Used settings to manage what you share across apps and platforms (N=664)	−.019	−.030	.060	−.048
	Shared particular kinds of information about yourself so that people won’t ask you about your real feelings or desires (N=660)	.14^e^	.137^e^	.153^e^	−.153^e^
**Internet rules that some people follow^g^**					
	Keep your computer in a public room (N=1355)	−.080^f^	−.085^f^	−.032	.039
	Remember people may not be who they say they are (N=1355)	.031	.035	.039	−.044
	Don’t use your real name online (N=1355)	−.056^d^	−.057^d^	−.053	.055^d^
	Don’t give out your address or phone number (N=1355)	−.112^e^	−.115^e^	−.08^f^	.089^f^
	Be careful with what you post online (N=1355)	−.083^f^	−.086^f^	−.066^d^	.071^f^
	Know how to block people online (N=1355)	−.011	−.016	.042	−.038
	None (N=1355)	.019	.014	.036	−.032

^a^Includes all respondents who reported sending sexts in any form.

^b^Includes all respondents who reported receiving sexts in any form.

^c^2014 data.

^d^Correlation is significant at the .05 level (2 tailed).

^e^Correlation <.001 (2 tailed).

^f^Correlation is significant at the .01 level (2-tailed).

^g^2012 data.

## Discussion

### Research Summary

This is the first Australian study to examine sexting behaviors using 2 representative random samples of young people. The research presents changes in sexting prevalence, beliefs about sexting, predictors of sexting, and the application of ICT safety skills by young people who engage in different types of sexting activities. Importantly, the results are examined using 4 sexting categories to include nonsexters, receivers of sexts, senders of sexts, and two-way sexters, which is recommended as best practice when conceptualizing sexting [17].

### Sexting Prevalence

The research found that from 2012 to 2014, not engaging in any form of sexting reduced significantly from one-third to a quarter of respondents. Meaning that three-quarters of young Australians had recently engaged in, or been exposed to, some form of sexting activity by 2014. This prevalence is high when compared with other Australian research using nonrepresentative or convenience samples [6,7,11,12].

By 2014, there was a significant increase in two-way sexting—with approximately 2 in 5 young people reporting that they had sent and received sexts. In the Australian sexting literature, this is the only known research to report on the prevalence of young people engaging in two-way sexting, so no direct comparisons can be made. When considering why so many young people are two-way sexting, research has reported that most young people share sexts within a dating relationship [17,34]. Therefore, it is possible that this finding reflects the reciprocal nature of sexting between sexual or presexual partners. Indeed, sexting today is increasingly viewed as a part of normal developmental behavior between young people [14,35].

Young people reporting only sending sexts remained minimal (<3%) between 2012 and 2014, whereas those only receiving sexts significantly rose—with almost one-third of young people experiencing this by 2014. Although young people were not directly asked the reasons for only receiving sexts, it is possible that this rise reflects that more young people in 2014 were not reciprocating sexting despite being sent sexts intended for them. Another more widely reported explanation in the literature is that young people may have received photos that were originally intended for someone else (ie, the sext had been forwarded to them) [17].

### Sociodemographic Predictors of Sexting

Previous research has predominately reported that young people in committed relationships are more likely to sext [34,36-38], whereas associations between gender and sexting have yielded mixed findings [16]. Some research examining interactions between both argue that relationship status, compared with gender, is significantly better at explaining interactions with sexting [34,39]. In this research, we examined 4 types of sexting activities and found that after adjusting for all variables, both gender and relationship status were associated with sexting. Specifically, two-way sexting, sending sexts, and receiving sexts were significantly associated with being male, whereas not sexting was associated with being female. In addition, two-way sexting and sending sexts were associated with relationship status. An explanatory reason for our findings can be drawn from previous sexting literature, which has reported that sexting can be socially riskier for certain individuals, such as females and those who are single [36]. These groups report stronger negative expectancies about sending and receiving sexts. Furthermore, the abovementioned secondhand sexting research has suggested that forwarded sexts can result in bullying or reputational damage of the individual who sent the original sext, particularly for young women [24,40-42], and young men are more likely to receive secondhand sexts [17]. This idea is further supported in our research findings that 78% of respondents believed that “females have to worry about messages being viewed by someone other than the person they had originally intended it for, more than males do,” whereas only 30% of them endorsed the opposite viewpoint.

Individuals who spoke a second language had elevated odds of sending sexts and two-way sexting. Other research reports that being from a racial and ethnic minority is associated with sexting [16,43-45], although this may vary by the individual’s ethnic background [46]. An additional finding from this study concerning the family composition was that young people who lived at home with their parents were less likely to receive sexts and more likely to be nonsexters, as compared with those who lived out of home. To our knowledge, this variable has not been previously researched [1]. However, the finding could be related to parental involvement—for example, parental restriction of mobile use has been previously found to be associated with lower sexting among young people [47]. Following this rationale, it is likely that those who live at home are more exposed to parental guidance and restrictions on their technology use and thus have less opportunity to sext.

### Health and Well-Being Predictors of Sexting

In the literature, although the research is scant, sexting has been associated with a greater likelihood of contemplated or attempted suicide [16] and suicidal ideation [48]. In this study, after adjusting for multiple sociodemographic, health and well-being, and ICT risk behaviors, two-way sexting and sending sexts were significantly associated with reporting suicidal thoughts and behaviors in the past 12 months. This research is neither able to demonstrate a causal relationship among variables nor can it determine whether sexting is an antecedent or result of suicidal thoughts and behaviors. However, an explanatory rationale for this is that sexting is a *risk behavior* for young people [49,50]. Previous research, for example, has drawn significant links between sexual risk behaviors, such as unprotected sex, and suicidal ideation and behaviors [51,52]. Another possible explanation is that young people experiencing mental health issues may sext to feel wanted [16]. Conversely, other researchers have suggested that both the lack of control over a sext once it is sent and possible pressure to sext when in relationships may contribute to psychological distress [17]. Indeed, in this study, the vast majority of respondents thought young people sexted as they “feel pressured to by friends or a dating partner” and that “messages usually end up being seen by more than just those to whom they were sent.”

Another well-being factor that the sexting literature has implicated is body image—with young people using sexting as a vehicle for obtaining feedback and reinforcing their body image [53,54]. This process of body image reinforcement has been cited as one of the major motivations for engaging in consensual sexting. However, research including body image concerns as a predictor of sexting is lacking. This research adds to the sexting literature by showing that body image concerns are a significant predictor of both two-way sexting and sending sexts. Some female-focused research has emphasized that sexual objectification of young women in general (ie, not digitally per se) is associated with depression, low self-esteem, eating distress, and negative body image [55,56]. This study suggests that body image may be a concern for both males and females who engage in 2two-way sexting and only sending sexts, as body image remained significant even after adjusting for all other variables including gender. Possible explanatory factors as to why young people with body image concerns have higher rates of sending sexts comes from research by Bianchi et al [54], who link young people with elevated body objectification with greater anxiety around sexuality and sexual intercourse. They argue that sexting may offer a way for these young people to experience sexuality despite their body-related concerns as it provides greater body image control, allows the sender to disengage emotionally, and be more assertive. Conversely, the same research acknowledges that body image–related motivations for sending sexts could expose a young person to suffer Web-based bullying and cyber victimization [54], which can exacerbate body image concerns.

### Information and Communication Technology Risk Predictors of Sexting

Although late-night internet use is a key risk factor for problematic internet use [57], previous research has not to our knowledge examined its association with sexting. In this research, all types of sexting activity (two-way, sending and receiving) were significantly associated with late-night internet use. Scholars argue that the technology capabilities of the smartphone, which enable *the selfie* combined with late-night use, make it easier than ever for young people “to cross the line from selfie to sext” [58]. Similarly, cyberbullying has also been reported to peak in frequency during the evenings [59].

Previous research has reported that those who engaged in sexting were more likely to experience cyber victimization [21]. This study extends this literature as it demonstrates that even after adjusting for all variables, receiving a sext is associated with being cyberbullied, and two-way sexting and sending sexts is significantly associated with cyberbullying others—whereas being a nonsexter is associated with reduced odds of being cyberbullied. Generally, research suggests that sexting can transform into cyberbullying when the sext is shared by the receiver without the sender’s consent [60]. In this study, it was the respondents who were more likely to be sending sexts (two-way and sending) that were engaging in cyberbullying. It is acknowledged that in this research, the survey did not distinguish between consensual sexting between intimate partners and nonconsensual sexting (such as sending secondhand sexts), which may influence the findings—particularly as relationship status predicted 2two-way sexting and sending sexts. Nevertheless, whether the sexts themselves form part of how respondents defined their cyberbullying experience is unknown, and further investigation is warranted—particularly given the link with serious concerns, including suicidal thoughts and behaviors.

### Sexting and Information and Communication Technology Safety Practices

Young people in this study who are sexting (two-way, sending, and receiving) appear to engage in more post hoc ICT safety behaviors. For example, correlations demonstrated that respondents who sexted were more likely to have tried to remove content they had posted and ignore threatening or offensive behavior toward themselves or others. Young people who sexted (two-way, sending and receiving) were also less likely to endorse preventative ICT safety strategies, including being careful about what they post on the Web and protecting their identity by not providing others on the Web with their real name, address, and phone number. Interestingly, nonsexters were significantly less likely to engage in many of the ICT safety items personally but were more likely to endorse protecting their identity and being careful about what they post on the Web. This may suggest that nonsexters are exposing themselves less to risky behaviors on the Web and are thus less likely to need to personally protect themselves using ICT safety strategies. Whether this is because they have received better ICT safety education is unknown, but it does appear that their beliefs about protecting themselves when on the Web are more in line with ICT safety education practices, compared with those who do engage in sexting.

### Implications for Policy and Practice

The research highlights the reality that there is a large proportion of young Australians sexting in some capacity. Although in Australia legal outcomes vary by state and territory and are addressed on a case-by-case basis [61], legal reforms in New South Wales have been implemented to reflect the view that sexting may be a normal part of sexual development and experimentation among many young people. Specifically, as a result of the Royal Commission into Institutional Responses to Child Sexual Abuse in 2018 [8], a legal exception has been introduced for children under 18 years who take, share, or possess nude photographs of themselves and others to minimize the risk of consenting children being convicted of possessing child pornography.

As sexting among young people is now more widespread, countries such as the United Kingdom have highlighted that a harm minimization approach, rather than an abstinence approach, may be the most appropriate path forward [62]. There have been calls in Australia to shift the sexting conversation, from ones focused predominantly on *risk* and prevention to one that focuses on ethics, respect, and responsibility [63]. The importance of educating young people about what it means to be an ethical user and consumer of technology is underscored [64]. Ensuring that young people’s views are incorporated, and their agency and decision making is respected is important [65], particularly as the voices of young people themselves are often ignored in the development of sexting guidelines and educational responses, despite this being a crucial step in ensuring that such initiatives are appropriate and relevant [66-68].

In line with other research [68], these results emphasize that sexting needs to be positioned within a wider conversation that involves in-depth exploration on topics such as consent, trust, gender, culture, psychological health, body image, and cyberbullying in the context of both technology and social media. Of particular importance relating to this research is sexting’s association with suicidal thoughts and behaviors and body image concerns. Currently, the Australian eSafety Commissioner’s lesson plans for teachers do not directly discuss with students how psychological and emotional well-being interacts with sexting [69]. Instead, there is greater focus placed on the potential social and legal consequences of sexting. In future, the sexting dialogue could be enriched by acknowledging and supporting young people, and those around them, in understanding and examining ways to address these issues. It should be emphasized that this is not solely engaging in risk-focused conversations but a proactive and in-depth dialogue exploring how young people can look after their own and each other’s physical, social, and emotional well-being on the Web.

The associations between sexting and other variables, including living with parents and late-night internet use, found in this research may demonstrate the important role parents and guardians play. However, parental monitoring of Web-based activities and handling conversations relating to sexting requires care and consideration—particularly as reviews of the digital safety literature [70] suggest that monitoring has the potential to force young people into becoming secretive in their online behaviors if not handled appropriately. Indeed, young people who sexted (two-way and sending) were less likely to endorse keeping a computer in a public room. Overall, a balance between safe sexting and independence needs to be found, and a focus should be placed on arming young people with ICT safety skills, particularly for when they no longer live at home. Both education and the cultivation of open, honest lines of communication with young people are seen as a crucial step in promoting ICT safety [70]. A challenge lies in the fact that research has implicated parents’ and teachers’ perceived lack of knowledge and skill relating to digital technologies as a barrier to meaningful conversations about sexting with young people [68]. Young people themselves have emphasized that parents also require greater educational support around sexting [68]. In line with previous research [71], providing support to adults so they can feel more confident in guiding young people through their cyber interactions is recommended. Online programs and apps may perform a key role in reducing the harms associated with sexting by empowering young people and adults to have better conversations around safe sexting and digital safety. These apps and electronic tools can be designed for specific groups of people. For example, apps such as the Australian Multicultural Foundation’s CyberParent [72] provide culturally and linguistically diverse parents with digital safety tools.

### Strengths and Limitations

There were numerous strengths of the study, which included the surveys comprising large Australia-wide randomly selected stratified samples representative of gender, age groups, and geographical location across 2 time points. The research captured the sociodemographics, mental health, and risk behaviors of young people. Validated measures for psychological distress and suicidal thoughts and behaviors were used, which is often a limitation of sexting research [73]. The sexting surveys captured more in‐depth questions relating to the direction (two-way, sending, receiving, and none) and the types of sexts sent (eg, talking about acts of a sexual nature and sending photos or videos showing yourself nude or nearly nude). These factors are especially noteworthy given that previous Australian research has relied on convenience and self-selecting samples and has demonstrated inconsistencies in definitions and the measurement of sexting behaviors [6,7,11,12]. Indeed, recent systematic reviews have concluded that the lack of a uniform definition of sexting is a problem that severely limits generalizability between studies [74]; by separating the direction and type of sexting, our research addressed this common concern.

However, the results demonstrated that three-quarters of young Australians had recently engaged in, or been exposed to, some form of sexting activity—which is high when compared with other Australian research [6,7,11,12]. This high prevalence rate may be a function of these CATI surveys including numerous sexting items, which is a more inclusive approach compared with single-item measures that comprise both sending and receiving sexts into the 1 variable. When compared with other Australian research, which used convenience samples (conducted at similar time points to these CATIs), this higher sexting prevalence rate may be attributed to a social desirability effect to some extent. Our previous research has shown that items that are more sensitive in nature, such as sexting, are more prone to underreporting in the presence of a telephone interviewer compared with online [75]. It is highly probable that the presence of a face-to-face interviewer in previous research [6,12] may further compound this social desirability effect, whereby respondents may minimize endorsement of embarrassing or unpleasant disclosures to maximize social acceptability and respectability as compared with a telephone interview. Hence, the rates presented in this paper may be a more accurate reflection of sexting prevalence.

Limitations in terms of survey length restricted the number of in‐depth questions that could be asked; for example, we did not explore consensual versus nonconsensual sexting. Furthermore, in our examination of reasons for sexting and beliefs about sexting, we analyzed *all senders* and *all receivers* of sexts rather than *senders only* and *receivers only* —which resulted in a substantial overlap between two-way sexters and senders because of the small *sender only* sample size (2014, n=20). This must be taken into account when interpreting results. When doing so, the results still provide highly useful comparisons between all types of sexting activity (two-way, sender, receiver, and no sexting).

Another research limitation is that we asked about sexting behaviors over the past year; although this is arguably not as problematic as asking respondents to report lifetime sexting, the lengthy period may produce issues with respondent recall. However, as highlighted in previous research [76], shorter periods that gauge current (30‐day) sexting can be problematic as it is an insufficient period to assess the full impact of sexting—such as possible mental health consequences.

### Conclusions

Our research clearly demonstrates that the majority of young Australians sext or are exposed to sexting. Trends over time suggest that the phenomenon of sexting is unlikely to go away. How a young person navigates this brave new world of sexual relationships in this digital landscape is complex, particularly as this research found sexting to be associated with negative health and well-being concerns, including suicidal thoughts and behaviors, body image issues, and cyberbullying. How the community works in partnership with young people to address this in future needs to be a multifaceted approach where sexting is positioned within a wider proactive conversation about gender, culture, psychosocial health, and respecting and caring for each other on the Web.

## Appendix

Multimedia Appendix 1 Sociodemographic and well-being changes from 2012 to 2014.

Multimedia Appendix 2 Crude adjusted odds ratios and adjusted odds ratios of the association of demographic, health and well-being items, and internet use with sexting activity (2014 data).

## Abbreviations

AOR: adjusted odds ratio
BMC: Brain and Mind Centre
CATI: computer-assisted telephone interviewing
CRC: Cooperative Research Centre
ICT: information and communication technology
RDD: random digit dialing
