# Supplementary material for: Sexting, Web-Based Risks, and Safety in Two Representative National Samples of Young Australians: Prevalence, Perspectives, and Predictors
Source: JMIR Ment Health. 2019 Jun 17;6(6):e13338. doi: 10.2196/13338 (PMC6601255; doi:10.2196/13338)
Supplement: Multimedia Appendix 1 [file mental_v6i6e13338_app1.pdf]

Multimedia Appendices

Multimedia Appendix 1.

Socio-demographic and wellbeing changes from 2012 to 2014

Item		2012	2014	X^2	*P*
Socio-demographic Items					
Gender	N	1400	1400	0.04	0.85
Male	%	50.0	50.4		
Female	%	50.0	49.6		
Age	N	1400	1400	0.42	0.81
16-18	%	34.6	35.6		
19-21	%	33.3	33.1		
22-25	%	32.1	31.2		
Language	N	1400	1400	0.93	0.34
English only	%	77.6	79.1		
Additional language	%	22.4	20.9		
Location	N	1400	1400	0.002	0.97
Major city	%	74.8	74.9		
Regional, rural or remote	%	25.2	25.1		
Living with parents	N	1400	1400	1.27	0.26
Yes	%	73.6	75.5		
No	%	26.4	24.5		
Main activity: Education	N	1399	1398	0.04	0.84

Yes	%	58.2	57.8		
No	%	41.8	42.2		
Main activity: Employment	N	1399	1398	0.55	0.46
Yes	%	35.2	33.9		
No	%	64.8	66.1		
Aboriginal and/or Torres Strait Islander	N	1399	1396	4.31	.04
Yes	%	2.1	3.4		
No	%	97.8	96.6		
Mental Health and Wellbeing Items					
Psychological distress	N	1400	1373	0.67	0.01
Low to moderate	%	79.1	74.9		
High to very high	%	20.9	25.1		
Suicidal thoughts and behaviours	N			.36	.55
Yes	%	18.3	17.4		
No	%	81.7	82.6		
Personal concern: Mental health or behavioural diagnosis	N	1389	1394	2.35	0.13
Yes	%	21.5	23.9		
No	%	78.5	76.1		
Personal concern: Alcohol or substance use diagnosis	N	1397	1400	7.25	0.007
Yes	%	97.5	98.9		
No	%	2.5	1.1		

Personal concern: Coping with stress	N	1390	1396	12.39	<.0001
Yes	%	58.5	65.0		
No	%	41.5	35.0		
Personal concern: Body image	N	1394	1393	20.49	<.0001
Yes	%	39.2	47.7		
No	%	60.8	52.3		
Personal concern: Depression	N	1393	1388	8.11	0.004
Yes	%	30.6	35.7		
No	%	69.4	64.3		
Personal concern: Bullying or emotional abuse	N	1397	1394	25.20	<0.0001
Yes	%	79.2	70.9		
No	%	20.8	29.1		
Personal concern: Alcohol	N	1394	1394	37.01	<0.0001
Yes	%	10.7	18.9		
No	%	89.3	81.1		
Personal concern: Drugs	N	1397	1399	42.46	<0.0001
Yes	%	8.2	16.2		
No	%	91.8	83.8		
Personal concern: Self-harm	N	1394	1396	37.00	<0.0001
Yes	%	8.1	15.5		
No	%	91.9	84.5		
Social support	M (SD)	-	9.88 (2.55)	-	-
Resilience	M (SD)	16.02 (2.82)	15.51 (2.62)	4.90[a]	0.001

Internet Use Items					
Use the Internet	N	1400	1399	.36	0.55
Yes	%	99.0	99.2		
No	%	1.00	0.8		
Internet access	N	1386	1388	19.41	<.0001
Every day or almost every day	%	95.3	98.0		
Not every day / almost every day	%	4.7	2.0		
Internet use after 11pm at night	N	1383	1387	3.41	0.07
Yes	%	63.1	66.4		
No	%	36.9	33.6		
Time spent on the internet	M (SD)	3.42 (2.93)	4.45 (3.14)	-8.93[a]	<.0001

a. t statistic
