# Supplementary material for: Sexting, Web-Based Risks, and Safety in Two Representative National Samples of Young Australians: Prevalence, Perspectives, and Predictors
Source: JMIR Ment Health. 2019 Jun 17;6(6):e13338. doi: 10.2196/13338 (PMC6601255; doi:10.2196/13338)
Supplement: Multimedia Appendix 2 [file mental_v6i6e13338_app2.pdf]

Multimedia Appendix 2.

Crude ORs and AORs of the association of demographic, health and wellbeing items, and internet use with sexting activity (2014 data)

	Sexting activity vs. all others[b] Exp(B) 95% CI							
**Variable**	**Two-way**		**Sender[c]**		**Receiver[d]**		**No-sexting**	
	**Crude OR 95% CI**	**AOR Exp(B) 95% CI**	**Crude OR 95% CI**	**AOR Exp(B) 95% CI**	**Crude OR 95% CI**	**AOR Exp(B) 95% CI**	**Crude OR 95% CI**	**AOR Exp(B) 95% CI**
Demographics								
Gender (male vs female)	0.61** [0.50-0.76]	0.63* [0.41-0.96]	0.61** [0.49-0.76]	0.58* [0.38-0.89]	0.87 [0.69-1.11]	0.59* [0.37-0.95]	1.17 [0.92-1.49]	1.73* [1.07-2.80]
Age (16-18yrs vs 19-21yrs)	1.65** [1.27-2.14]	1.13 [0.71-1.80]	1.64** [1.27-2.13]	1.11 [0.70-1.76]	1.19 [0.89-1.57]	0.83 [0.51-1.37]	0.78 [0.58-1.05]	1.22 [0.73-2.04]
Age (16-18yrs vs 22-25yrs)	2.07** [1.59-2.70]	1.34 [0.78-2.30]	2.11** [1.62-2.75]	1.23 [0.72-2.10]	1.23 [0.92-1.64]	0.72 [0.41-1.27]	0.84 [0.63-1.11]	1.56 [0.87-2.79]
English only language spoken (No vs Yes)	1.23 [0.95-1.61]	1.66* [1.01-2.73]	1.21 [0.93-1.58]	1.71* [1.04-2.80]	1.32* [1.00-1.76]	1.53 [0.93-2.55]	0.76 [0.57-1.01]	0.62 [0.37-1.03]
Indigenous (No vs Yes)	0.98 [0.55-1.75]	0.74 [0.23-2.37]	1.09 [0.62-1.96]	0.93 [0.30-2.94]	1.24 [0.63-2.47]	0.93 [0.23-3.80]	0.67 [0.32-1.39]	0.70 [0.14-3.65]
Location (major city vs regional, rural or remote)	0.93 [0.73-1.19]	1.01 [0.66-1.56]	0.96 [0.75-1.23]	1.00 [0.65-1.54]	1.05 [0.80-1.38]	0.97 [0.59-1.59]	0.91 [0.68-1.21]	1.05 [0.63-1.74]
Currently in education (No vs Yes)	0.51** [0.41-0.63]	0.72 [0.36-1.42]	0.51** [0.41-0.63]	0.80 [0.41-1.59]	0.67* [0.52-0.85]	0.66 [0.29-1.50]	1.55* [1.21-1.99]	1.32 [0.58-3.02]
Currently in employment (No vs Yes)	1.97** [1.57-2.47]	1.36 [0.68-2.74]	2.03** [1.62-2.54]	1.57 [0.78-3.16]	1.66** [1.27-2.16]	1.05 [0.45-2.45]	0.57** [0.43-0.74]	0.76 [0.32-1.81]
Live with parent(s) or guardian(s) (No vs Yes)	0.64** [0.50-0.82]	0.88 [0.54-1.44]	0.60** [0.47-0.76]	0.79 [0.48-1.28]	0.64* [0.48-0.86]	0.53* [0.29-0.93]	1.79** [1.32-2.43]	2.36* [1.28-4.36]
Currently in a relationship (No vs Yes)	2.43** [1.78-3.32]	2.16** [1.47-3.19]	2.32** [1.70-3.16]	2.11** [1.43-3.09]	1.57* [1.10-2.25]	1.14 [0.73-1.76]	0.66* [0.46-0.95]	0.90 [0.57-1.41]
Health and Wellbeing								
Psychological distress (K10: low/moderate vs high/very high)	1.04 [0.82-1.34]	0.68 [0.39-1.17]	1.06 [0.83-1.35]	0.67 [0.39-1.15]	1.86** [1.37-2.52]	1.36 [0.74-2.49]	0.51** [0.37-0.70]	0.72 [0.39-1.35]
Suicidal ideation and or acts (PSFS)	1.50* [1.13-2.00]	1.86* [1.00-3.46]	1.62* [1.22-2.16]	2.21* [1.19-4.10]	1.92** [1.34-2.76]	1.10 [0.53-2.33]	0.43** [0.30-0.65]	0.63 [0.28-1.41]
Mental health diagnosis (No vs Yes)	1.20 [0.94-1.54]	0.92 [0.57-1.51]	1.25 [0.97-1.60]	0.93 [0.57-1.52]	1.87** [1.37-2.54]	1.68 [0.96-2.96]	0.49** [0.36-0.68]	0.57 [0.32-1.02]

Alcohol or other substance misuse diagnosis (No vs Yes)	**4.17*<br>[1.34-13.00]**	3.74<br>[0.38-36.96]	**3.93*<br>[1.26-12.25]**	3.34<br>[0.33-33.52]	1.60<br>[0.45-5.64]	0.72<br>[0.07-7.76]	0.67<br>[0.19-2.38]	1.96<br>[0.17-21.99]
Personal concern: Alcohol (No vs Yes)	**1.35*<br>[1.03-1.76]**	1.04<br>[0.58-1.87]	1.28<br>[0.98-1.67]	1.00<br>[0.56-1.78]	**1.73*<br>[1.24-2.41]**	1.04<br>[0.51-2.11]	**0.61*<br>[0.44-0.86]**	1.02<br>[0.49-2.09]
Personal concern: Body Image (No vs Yes)	**1.60**<br>[1.29-1.98]**	**2.06*<br>[1.33-3.19]**	**1.56**<br>[1.27-1.94]**	**2.00*<br>[1.30-3.08]**	**1.92**<br>[1.51-2.46]**	1.44<br>[0.90-2.32]	**0.52**<br>[0.41-0.67]**	0.70<br>[0.43-1.14]
Personal concern: Bullying (No vs Yes)	**1.35*<br>[1.07-1.70]**	1.37<br>[0.80-2.34]	**1.28*<br>[1.01-1.61]**	1.16<br>[0.68-1.97]	**1.53*<br>[1.16-2.02]**	0.85<br>[0.46-1.58]	**0.69*<br>[0.52-0.92]**	1.53<br>[0.81-2.89]
Personal concern: Stress (No vs Yes)	1.13<br>[0.90-1.41]	1.11<br>[0.71-1.73]	1.13<br>[0.90-1.41]	1.16<br>[0.74-1.81]	**1.57**<br>[1.23-2.00]**	1.26<br>[0.78-2.04]	**0.63**<br>[0.49-0.81]**	0.75<br>[0.46-1.22]
Personal concern: Depression (No vs Yes)	**1.40*<br>[1.12-1.74]**	0.63<br>[0.35-1.12]	**1.41*<br>[1.13-1.76]**	0.68<br>[0.38-1.20]	**1.60**<br>[1.24-2.07]**	0.62<br>[0.33-1.16]	**0.63*<br>[0.46-0.79]**	1.56<br>[0.81-2.99]
Personal concern: Drugs (No vs Yes)	**1.86**<br>[1.40-2.48]**	1.85<br>[0.91-3.75]	**1.81**<br>[1.36-2.41]**	2.00<br>[0.99-4.05]	**2.17**<br>[1.49-3.16]**	2.39<br>[0.98-5.83]	**0.47**<br>[0.32-0.68]**	**0.35*<br>[0.14-0.90]**
Personal concern: Self-harm (No vs Yes)	**1.35*<br>[1.01-1.80]**	0.63<br>[0.31-1.25]	**1.34*<br>[1.00-1.79]**	0.62<br>[0.31-1.24]	**1.59*<br>[1.11-2.27]**	0.72<br>[0.32-1.64]	**0.62*<br>[0.43-0.89]**	1.45<br>[0.61-3.43]
Resilience (BRCS)	-	1.03<br>[0.96-1.12]	-	1.03<br>[0.95-1.11]	-	1.02<br>[0.94-1.11]	-	0.99<br>[0.90-1.08]
Social support (SSSS)	-	0.96<br>[0.88-1.04]	-	0.96<br>[0.88-1.04]	-	1.03<br>[0.93-1.13]	-	0.97<br>[0.88-1.07]
Internet use and online and communication risks								
Has been cyberbullied in the past 12 months (No vs Yes)	**2.02**<br>[1.52-2.69]**	1.11<br>[0.61-2.02]	**1.97**<br>[1.48-2.63]**	1.07<br>[0.59-2.94]	**3.33**<br>[2.17-5.10]**	**4.61*<br>[1.85-11.50]**	**0.29**<br>[0.19-0.46]**	**0.22*<br>[0.09-0.56]**
Cyberbullied others in the past 12 months (No vs Yes)	**3.25**<br>[2.11-5.01]**	**5.28**<br>[2.20-12.65]**	**3.21**<br>[2.08-4.97]**	**4.79**<br>[2.00-11.44]**	**2.65*<br>[1.46-4.80]**	1.40<br>[0.41-4.83]	**0.37*<br>[0.20-0.68]**	0.87<br>[0.25-3.02]
Average time spent on internet	-	1.02<br>[0.96-1.09]	-	1.02<br>[0.96-1.09]	-	0.98<br>[0.92-1.05]	-	1.02<br>[0.95-1.09]
Late night internet use (No vs Yes)	**2.01**<br>[1.59-2.54]**	**1.92*<br>[1.24-2.95]**	**2.03**<br>[1.61-2.56]**	**1.88*<br>[1.23-2.88]**	**2.12**<br>[1.66-2.71]**	**2.58**<br>[1.67-3.98]**	**0.45**<br>[0.35-0.58]**	**0.38**<br>[0.24-0.59]**

a. ** Correlation is significant at the 0.01 level (2-tailed); * Correlation is significant at the 0.05 level (2-tailed).
b. Cells left blank indicate scale items which do not have a crude OR calculation option.
c. Includes all respondents who reported sending sexts in any form.
d. Includes all respondents who reported receiving sexts in any form.
